# Sow performance in response to natural betaine fed during lactation and post-weaning during summer and non-summer months

**DOI:** 10.1186/s40104-020-00471-0

**Published:** 2020-07-02

**Authors:** S. M. Mendoza, R. D. Boyd, J. Remus, P. Wilcock, G. E. Martinez, E. van Heugten

**Affiliations:** 1 Department of Animal Science, North Carolina State University, Raleigh, NC 27695 USA; 2 The Hanor Company Inc., Franklin, KY 42134 USA; 3 DuPont Animal Nutrition, Wilmington, DE 19803 USA; 4 AB Vista, Marlborough, UK

**Keywords:** Betaine, Lactation, Post-weaning, Reproduction, Sows

## Abstract

**Background:**

Two studies were conducted to evaluate the effects of dietary natural betaine on sow reproductive performance during summer (Exp. 1) and non-summer months (Exp. 2). Treatments were designed as a 2 × 2 factorial arrangement with factors including dietary betaine (0 or 0.2%) and period of supplementation (lactation or post-weaning until 35 days post-insemination). In Exp. 1, 322 and 327 sows and in Exp. 2, 300 and 327 sows representing young (parity 1 and 2) and mature (parity 3 to 6) sows, respectively, were used.

**Results:**

In Exp. 1, supplementation of betaine during lactation increased sow body weight losses (− 11.95 vs. −14.63 kg; *P* = 0.024), reduced feed intake (4.12 vs. 4.28 kg/d; *P* = 0.052), and tended to reduce percentage of no-value pigs (*P* = 0.071). Betaine fed post-weaning reduced weaning-to-estrus interval (5.75 vs. 6.68 days; *P* = 0.054) and farrowing rate (86.74% vs. 91.36%; *P* = 0.060), regardless of parity group. Post-hoc analysis with sows clustered into 3 parity groups (1, 2 and 3, and 4+) indicated that betaine fed in lactation to parity 4+ sows (*P* = 0.026) and betaine fed post-weaning to parity 1 sows increased the number of pigs born in the subsequent cycle (*P* ≤ 0.05). In Exp. 2, betaine fed during lactation tended to reduce the weaning-to-estrus interval (6.64 vs. 7.50 days; *P* = 0.077) and farrowing rate (88.23% vs. 83.54%; *P* = 0.089), regardless of parity group. Feeding betaine post-weaning reduced number of pigs born (13.00 vs. 13.64; *P* = 0.04) and pigs born alive (12.30 vs. 12.82; *P* = 0.075), regardless of parity group.

**Conclusions:**

Using 0.2% betaine during the non-summer months did not benefit sow performance. During the summer, betaine supplementation in lactation increased subsequent litter size in parity 4+ sows. Betaine fed during the post-weaning period reduced the wean-to-estrus interval and farrowing rate, increased total number of pigs born for parity 1 sows and reduced total number of pigs born to parity 4+ sows. Further research is needed to determine if the detrimental effects on feed intake and farrowing rate may be correlated and depend on dietary betaine level.

## Background

Sow reproductive performance severely declines during the summer season. During the summer months sow non-productive days increase by 5 to 19 days [1] and this is mainly due to a delay in estrus, failure to return to estrus, or embryo losses after mating [2]. Farrowing rate has been reported to be 20% lower during the summer than sows bred during non-summer months [2]. In addition, total number of pigs born is estimated to be reduced by 0.05 piglets for each 1 °C above 20 °C on the day of insemination [3]. Above 25 °C, sows decrease feed intake by 0.5 kg/d per additional 1 °C [4] and increase tissue mobilization to sustain milk production [5]. Sows with poor feed intake during lactation continue the subsequent reproductive period with a negative energy balance [6], which acts as a negative feedback to prevent the onset of a new reproductive cycle.

Betaine, or tri-methyl glycine, is a methyl donor and osmoregulator that naturally accumulates in organisms that are adapted to saline or drought conditions [7, 8]. The role of betaine as an osmolyte has important implications because it can maintain cell volume and integrity under challenging conditions, including heat stress. Ramis et al. [9] fed betaine to sows during lactation and reported an improvement in litter gain. In addition, dietary betaine supplementation to lactating sows reduced the weaning to estrus interval [9–11] and increased subsequent litter size [10, 12]. van Wettere et al. [13] reported that feeding betaine to gestating sows during the summer increased litter size, in particular, in mature sows. The effect of betaine supplementation to sows after weaning, during breeding, and early in gestation has not been evaluated previously. Betaine may be beneficial to the embryo during implantation as an osmoprotectant in the initial cellular division, as a methyl donor in the blastocyst [14, 15] and by reducing plasma homocysteine [16].

We hypothesize that the osmolyte betaine can alleviate the negative effects caused by high temperature in sows, by reducing the weaning to estrus interval, and increasing farrowing rate and subsequent litter size. The objective of the current study was to evaluate the impact of dietary betaine fed during lactation and post-weaning as a heat abatement strategy in sows exposed to heat stress.

## Material and methods

Two studies were conducted in a commercial research facility in Oklahoma (2600 sows). Sows (Camborough, Pig Improvement Company (PIC), Hendersonville, TN) and piglets (TR-4 × Camborough product sows, PIC) used in these experiments were humanely treated following the practices outlined in the Guide for the Care and Use of Animals in Agricultural Research and Teaching [17]. Protocols were under the supervision of licensed veterinarians.

The two studies had the same experimental design and used the same diet formulations with the difference being the season that they were conducted. The design was a 2 × 2 factorial arrangement. Factors included dietary betaine (Vistabet® 96, AB Vista, Marlborough, UK) supplemented at 0 or 0.2% and 2 periods of supplementation (lactation or post-weaning until 35 days post-insemination). Dietary treatments were initiated immediately after farrowing and sows were fed lactation diets with either 0 or 0.2% betaine. After weaning, sows fed 0% betaine during lactation were either fed 0 or 0.2% betaine from weaning until 35 days post-breeding and, similarly, sows fed 0.2% betaine during lactation were fed either 0 or 0.2% betaine post-weaning until 35 days post-breeding. Diets were formulated to meet or exceed NRC [18] nutrient recommendations and manufactured by a commercial feed mill (Hanor Company, Enid, Oklahoma). Lactation diets (Table 1) were formulated to contain 3.31 g standardized ileal digestible (SID) lysine/Mcal ME, 0.56 SID methionine+cystine/lysine ratio, and 1.9 g of choline/kg of diet. Diets for the post-weaning period were formulated to contain 1.82 g SID lysine/Mcal ME, 0.69 SID methionine+cystine/lysine ratio, and 1.15 g of choline/kg of diet (Table 1). Diets were formulated to supply sufficient methyl donors to specifically evaluate the osmolyte properties of betaine. For both diets, the inclusion of betaine was made at the expense of corn. Diets were color-coded to visually confirm that proper diets were fed to the correct treatment groups. Feed samples were collected at the feed mill for every batch and every week at the farm to chemically verify the diets.

**Table 1:** Composition of the experimental diets, as fed basis^a^

Item	Diet^b^	
	Lactation	Post-weaning
Ingredient, %		
Corn, medium grind	45.915	47.025
Soybean meal, 46.5% CP	32.1	4.0
Rice bran	10	15
Wheat middlings	6	30
Poultry fat	2.35	0
Limestone	1.12	1.45
Monocalcium phosphate, 21% P	0.985	0.870
Potassium, magnesium sulfate^c^	0.50	0.50
Salt	0.40	0.40
Sow vitamin-mineral premix^d^	0.20	0.20
Choline chloride, 60%	0.125	0.125
Anti-caking aid^e^	0.100	0.100
Organic mineral source [Zn-Mn-Cu]^f^	0.075	0.075
*L*-Lysine	0.050	0.150
*L*-Threonine	0.035	0.075
*DL*-Methionine	0.015	0.000
Iron oxide^g^	0.030	0.030
Calculated nutrient composition		
ME, Mcal/kg	3.30	3.04
CP, %	21.17	12.90
Lysine, %	1.17	0.64
Standardized ileal digestible Lys, %	1.05	0.56
Standardized ileal digestible Lys/ME, g/Mcal	3.31	1.82
Standardized ileal digestible Met+Cys:Lys	0.56	0.69
Ca, %	0.82	0.85
Available P, %	0.40	0.44
Choline, g/kg	1.90	1.15

^a^Diets were formulated to exceed NRC (2012) requirements

^b^To create betaine added diets, natural betaine (Vistabet® 96, AB Vista, Malborough, UK) was added at 0.2% at the expense of corn

^c^Dynamate (Mosaic, Plymouth, MN). Added as a laxative

^d^Supplied per kg of complete diet: vitamin A, 11,023 IU; vitamin D_3_, 1764 IU; vitamin E, 51 IU; vitamin K, 4.4 mg; vitamin B_12_, 0.044 mg; riboflavin, 8.8 mg; d-pantothenate, 26.5 mg; niacin 55.1 mg; thiamine, 3.3 mg; pyridoxine, 3.3 mg; folic acid, 1.21 mg; biotin, 0.28 mg; Zn, 125 mg; Fe, 100 mg; Mn, 50 mg; Cu, 25.0 mg; I, 0.7 mg; Se, 0.3 mg; phytase, 661 FTU (Phyzyme, Danisco A/S, Copenhagen, Denmark), and chromium, 0.4 mg/kg

^e^Dry anti-caking aid and non-nutritive carrier (KALLSIL, Kemin Industries, Inc., Des Moines, IA)

^f^Supplied per kg of complete diet: Zn, 50 mg from Zn amino acid complex, Mn, 20 mg from Mn amino acid complex, and Cu, 10 mg from Cu amino acid complex (Availa, Zinpro Corporation, Eden Prairie, MN)

^g^Used to color code diets. Diet with 0% betaine contained Fe_3_O_4_ (black) and diet with 0.2% betaine contained Fe_2_O_3_ (red)

Proximate analysis (Table 2) of the diets was conducted by the Agricultural Experiment Station Chemical Laboratories, University of Missouri (Columbia, MO) using AOAC [19] procedures. Diets were analyzed for crude protein (Method 990.03), crude fat (Method 920.39a), crude fiber (Method 978.10), and ash (Method 942.05). Betaine concentrations of the diets were analyzed by the Bindley Bioscience Center at Purdue University (West Lafayette, IN) by liquid chromatography/mass spectrometry using an Agilent 1200 Rapid Resolution liquid chromatography system coupled to an Agilent 6460 series QQQ mass spectrometer (Agilent Technologies Inc., Santa Clara, CA).

**Table 2:** Analyzed nutrient composition of the experimental diets, as fed basis^a^

			Analyzed composition, %				
Diet	Experiment^b^	Added betaine, %	CP	Crude fat	Crude fiber	Ash	Betaine
Lactation	1	0.0	22.09	5.68	3.10	7.16	0.057
Lactation	1	0.2	19.34	4.82	3.28	6.20	0.197
Lactation	2	0.0	21.66	5.86	3.08	6.91	0.055
Lactation	2	0.2	21.25	6.01	3.20	6.89	0.347
Gestation	1	0.0	12.51	5.00	4.19	7.16	0.287
Gestation	1	0.2	12.81	5.42	4.13	6.96	0.489
Gestation	2	0.0	13.23	4.93	4.36	6.65	0.265
Gestation	2	0.2	13.26	5.37	4.27	6.75	0.508

^a^Proximate analysis was conducted by the Agricultural Experiment Station Chemical Laboratories, University of Missouri, Columbia, MO, USA. Betaine was analyzed by the Bindley Bioscience Center at Purdue University, West Lafayette, IN, USA

^b^Exp. 1 was conducted during summer months and Exp. 2 was conducted during non-summer months

For both experiments, sows were housed in the same farrowing rooms and the same breeding barn. Environmental temperatures in the farrowing rooms were set at 23.5 °C at farrowing and gradually reduced to 21 °C at weaning. Supplemental heat or cooling were provided using gas heaters and wet-cell cooling pads, respectively. Heat lamps were used to provide additional heat for piglets. In the gestation barn, environmental temperatures were set at 20 °C year-around and temperature was managed using gas heaters and fans for ventilation. During the course of the experiments, ambient temperature and humidity of the farrowing room and breeding barn were recorded using data recorders (logtag, MicroDAQ Ltd., Contoocook, NH, USA). Data recorders measured temperature and humidity every 5 min. For the farrowing rooms, a data logger was placed for each farrowing group. For the breeding barn, two loggers were placed, one on each side of the barn. A heat index was calculated considering air temperature and relative humidity, according to the US National Weather Service.

### Exp. 1

A total of 649 sows entered the farrowing room in groups of 20 to 26 sows. Within each group, sows were randomly assigned within parity groups to 1 of 4 treatments. The experimental design consisted of a generalized randomized complete block design and was balanced by parity. A total of 169, 153, and 327 sows representing parity 1 (P1), 2 (P2), and 3 to 6 (P3+) were used in the study. Each group of sows aimed to contain 50% of young sows (P1 and P2) and 50% of mature sows (P3+). The first group entered the farrowing room in June and the last group (30^th^) was weaned in September. The first group was weaned in July and the last group was relocated to the gestation barn in November (day 35 post-insemination, end of dietary supplementation treatments).

### Exp. 2

A total of 627 sows entered the farrowing room in groups of 22 to 24. Within each group, sows were randomly assigned to 1 of 4 treatments considering parity groups. A total of 250, 50, and 327 sows representing P1, P2, and P3+ were used in the study. Each group of sows aimed to contain 50% of young sows (P1 and P2) and 50% of mature sows (P3+). The first group entered the farrowing room in February and the last group (28^th^) was weaned in June. The first group was weaned in March and the last group was relocated to the gestation barn in July (day 35 post-insemination, end of supplementation treatments).

### Lactation period

Sows were weighed individually when they entered the farrowing room. In Exp. 1, sows entered the farrowing room on day 109 ± 1 and in Exp. 2 on day 112 ± 2 of gestation. Between placement and farrowing, sows were fed 1.82 kg/d of a common lactation diet without betaine. After farrowing, dietary treatments were provided, and feed additions and feed refusals were recorded for each sow until weaning. Sows were fed to satiety and feed was offered at 08:00 h and 14:00 h to appetite, ensuring sows had some feed in front of them at all times. Number and weight of pigs at birth (alive, stillborn, and mummies) were recorded and placenta and weight of fluids were calculated from the equations reported by Walker and Young [20]. Litter birth weight, estimated placenta weight and estimated weight of fluids were subtracted from the weight of the sow at placement to estimate sow weight at farrowing. Sows were weighed for a second time on day 21 of lactation. The difference between sow weight on day 21 and farrowing weight represented the body weight (BW) change (loss or gain) during lactation. Sow gain:feed was calculated as total gain during lactation (sow BW gain or loss plus total litter gain) divided by total feed intake during lactation.

Piglet cross-fostering was done 18 to 24 h after farrowing to allow for colostrum intake from their mothers. Cross-fostering was done within litters that were assigned to the same treatment. Litter size was standardized to 12 pigs. Handling, processing, and vaccination of piglets was performed according to the recommendations of licensed veterinarians and were identical for all litters. Litter weight gain was calculated as the difference between the weight of the litter on day 21 and the weight of the litter after cross-fostering (Exp. 1, *n* = 649 litters; Exp. 2, subset *n* = 352 litters). Date and weight of dead piglets were recorded. The piglets that did not reach 3.62 kg of BW on day 21 were considered no-value pigs. Pigs did not receive creep feed or milk replacer during the experiment. Piglets were weaned at the first opportunity after day 21 following the production schedule (lactation days 22 ± 1 in Exp. 1 and 23 ± 1 in Exp. 2).

Rectal temperature and respiration rate were measured on day 18 of lactation in a subset of sows (*n* = 44 in Exp. 1 and *n* = 64 in Exp. 2). Measurements were taken between 16:00 h and 18:00 h. Rectal temperature was measured using a digital thermometer (M750 Series, GLA Agricultural Electronics, San Luis Obispo, CA, USA). Respiration rate was measured as the number of flank movements per 30 s and was reported on a per min basis.

### Post-weaning to 35 days post-insemination

The breeding barn at the sow facilities is equipped with drop feeders (capacity of 3.62 kg) and has two independent feeding systems. One-half of the barn received the 0% betaine and the other half the 0.2% betaine post-weaning diets. After weaning, sows were distributed in the barn according to their assigned treatment and maintained until day 35 post insemination. Sows had ad libitum access to feed before signs of estrus were detected. At that point, sows were artificially inseminated and feed drops were adjusted to offer 1.8 to 2.7 kg of feed depending on sow body condition. Feed was delivered in two meals during the day. In Exp. 1 feed was delivered at 02:00 h and 08:00 h, and in Exp. 2 feed was delivered at 09:00 h and 12:00 h. The schedule followed these recommendations to avoid high temperatures during the summer months at the time feed was provided.

Estrus detection and artificial insemination were performed following the standard operating procedures of the farm. Sows that did not return to estrus within 14 days discontinued the dietary treatments and were not used in the final analysis for subsequent litter size. After day 35 post-insemination, sows were moved to the gestation barn and were fed the standard gestation diet (control without betaine). Data collection during this period included days to estrus, the number of sows bred, the number of sows bred within 14 days after weaning, sows that farrowed, and sows that were culled. Once sows reached approximately day 110 of gestation, they were moved to the farrowing room and subsequent litter size at birth was recorded (pigs born alive, stillborn pigs, and mummies). Rectal temperature and respiration rate were measured on day 12 of gestation in a subset of sows (*n* = 44 in Exp. 1 and *n* = 64 in Exp. 2) using the same procedures as described for the lactation period.

### Statistical analysis

Sow and litter performance data were analyzed using the MIXED procedure of SAS (SAS Inst. Inc., Cary, NC). The model included the effect of betaine in lactation, parity group, and their interaction as fixed effects. The groups of sows (Exp. 1 groups = 30; Exp. 2 groups = 28) that entered the farrowing room together were considered as the random effect. Rectal temperature and respiration rate were analyzed using the MIXED procedure of SAS (SAS Inst. Inc., Cary, NC). The model included the effect of betaine in lactation, parity group, and their interaction as fixed effects. The sow was considered as a random effect.

Subsequent reproductive performance data were analyzed using the MIXED procedure of SAS for days to estrus, total pigs born, pigs born alive, stillborn pigs and mummies. The GLM procedure of SAS was used for dichotomous variables. The model included the effect of betaine during lactation, the effect of betaine post-weaning, parity group, and their interaction as fixed effects. Groups of sows were considered as a random effect for days to estrus, total pigs born, pigs born alive, stillborn pigs, and mummies. A *post-hoc* analysis was conducted for Exp. 1 to further separate the parity groups into parity 1, parity 2 and 3, and parity 4, 5, and 6 to determine the impact of betaine within these parity groups, using similar models as previously described. Least squares means were reported, and differences were considered statistically significant at *P* ≤ 0.05 and were considered tendencies when 0.05 < *P* ≤ 0.10. The least significant difference method was used for single degree of contrast multiple comparisons.

## Results

Chemical analysis of the diets was in reasonable agreement with calculated concentrations. Specifically for betaine, the concentration of betaine in control lactation diets averaged 0.056% and was 0.272% for the betaine supplemented diets, representing 0.216% additional betaine relative to the control diets. The concentration of betaine in the control gestation diets was 0.276% compared to 0.498% in the betaine supplemented diets, representing 0.222% added betaine.

### Exp. 1

The average temperature of the farrowing room during the lactation period was 25.4 ± 2.3 °C (minimum of 16.4 and maximum of 35.7 °C) and relative humidity was 72.5% ± 7.4% (minimum of 37.4% and maximum of 95.1%), resulting in a heat index of 27.0 ± 3.1 °C (minimum of 17.8 and maximum of 41.2 °C). Sows were above an environmental temperature of 26 °C 48.9% of the time and were above a heat index of 26 °C for 62.3% of the time. The average temperature of the breeding barn (during the post-weaning until 35 days post-insemination) was 23.3 ± 2.9 °C (minimum of 16.8 and maximum of 31.2 °C) and relative humidity was 72.1% ± 8.6% (minimum of 40.9% and maximum of 93.2%), with a calculated heat index of 24.9 °C (minimum of 18.0 and maximum of 36.6 °C). Sows were above an environmental temperature of 26 °C for 19.1% of the time and above a heat index of 26 °C for 28.0% of the time.

As expected, P3+ sows had greater BW at placement, after farrowing, and at day 21 of lactation (*P* < 0.001; Table 3). Losses in BW during lactation were greater for P1 and P2 sows than P3+ sows (*P* < 0.001). Average daily feed intake and sow gain:feed were greater for P3+ sows (*P* < 0.004). Litter gain was greater for piglets from P3+ sows (*P* < 0.001), but no differences in the number of pigs weaned or mortality were observed due to parity group (*P* ≥ 0.385). Supplementation of betaine during the lactation period increased BW losses (*P* = 0.024) and this was related to lower feed intake (*P* = 0.052). No differences in litter gain (*P* = 0.350) or the number of pigs weaned were observed (*P* = 0.535) due to betaine supplementation. However, supplementation of betaine tended to reduce the percentage of no-value pigs (*P* = 0.071).

**Table 3:** Effect betaine fed during lactation on sow and litter performance during the summer months (Exp. 1)^a^

	Parity 1 and 2		Parity 3+		SEM	*P*-values^b^	
	Betaine, %						
Item	0	0.2	0	0.2		Betaine	Parity group
Sows, *n*	159	163	165	162			
BW at placement, kg^c^	246.4	247.2	278.9	280.9	2.143	0.443	< 0.001
BW at farrowing, kg^d^	227.1	227.3	257.7	257.5	1.966	0.983	< 0.001
BW at day 21, kg	209.1	206.2	251.8	249.2	1.898	0.148	< 0.001
BW change, kg	−18.0	−21.0	− 5.9	−8.2	1.399	0.024	< 0.001
Sow BW gain, kg/d	−0.856	−1.002	−0.283	−0.391	0.067	0.024	< 0.001
Lactation length, d	22.10	22.09	22.16	22.22	0.101	0.748	0.245
Feed intake, kg/d	3.687	3.414	4.873	4.823	0.094	0.052	< 0.001
Sow gain:feed^e^	0.428	0.388	0.475	0.464	0.022	0.224	0.004
Litter weight after cross-fostering, kg^f^	17.90	18.14	18.45	18.76	0.225	0.164	0.004
Litter weight on day 21, kg	68.9	67.2	71.5	72.0	0.879	0.431	< 0.001
Litter gain, kg	52.7	51.1	54.8	55.2	0.727	0.350	< 0.001
Pigs weaned per litter	11.02	10.94	10.92	10.90	0.097	0.535	0.432
Piglet mortality, %	8.08	8.73	8.92	9.15	0.804	0.548	0.385
No-value pigs, %^g^	2.30	1.81	2.12	1.38	0.370	0.071	0.368

^a^Values represent least squares means of n sows for sow and litter performance. The experiment was conducted from June to September

^b^The statistical analysis tested for main effects of betaine supplementation, parity group and their interaction. Group of placement (1 to 28) was used as random effect. No significant interactions between parity group and betaine supplementation were detected (*P* ≥ 0.13)

^c^Sow body weight (BW) was measured prior to sows entering the farrowing room (109 ± 1 day of gestation)

^d^Sow BW after farrowing was calculated using equations of Walker and Young [20]

^e^Sow gain:feed was calculated as sow BW gain or loss plus total litter gain divided by total feed intake during lactation

^f^Litters were standardized to 12 pigs per litter

^g^No value pigs consisted of pigs weighing less than 3.62 kg at day 21 of lactation

The average daily temperature of the farrowing room was 28.1 °C (range of 27.3 to 28.7 °C), humidity averaged 72.9% (range of 68.3% to 77.4%) and the calculated heat index was 31.2 °C (range of 30.0 to 32.5 °C) when rectal temperature and respiration rate were measured in a subset of sows. Sows were above an environmental temperature and heat index of 26 °C for 100% of the time when measurements were taken during lactation. During the post-weaning period, environmental temperature was 27.2 °C (range of 24.2 to 29.2 °C), humidity was 63.4% (range of 53.7% to 75%), and the heat index was 28.7 °C (range of 25.1 to 31.2 °C). Sows experienced an environmental temperature and heat index over 26 °C for 91.3 and 94.5% of the time when respiration rate and rectal temperatures were measured. Betaine supplementation in lactation or post-weaning did not affect (*P* ≥ 0.091) rectal temperature or respiration rate (Table 4).

**Table 4:** Effect of betaine supplementation during lactation and post-insemination on rectal temperature and respiration rate^a^

	Parity 1 and 2		Parity 3+		SEM	*P*-values^b^		
	Betaine, %					Betaine	Parity group	Interaction
Item	0	0.2	0	0.2				
Summer (Exp. 1)^c^								
Lactation								
Rectal temperature, °C	39.54	39.44	39.56	39.56	0.127	0.693	0.597	0.718
Respiration rate^d^	65.77	62.58	81.90	74.89	8.684	0.562	0.110	0.827
Post-insemination								
Rectal temperature, °C	38.53	38.49	38.46	38.53	0.076	0.825	0.851	0.470
Respiration rate^d^	22.00	18.58	13.20	12.33	2.031	0.399	0.005	0.614
Non-summer (Exp.2)^e^								
Lactation								
Rectal temperature, °C	39.41	39.43	39.19	39.25	0.138	0.772	0.154	0.889
Respiration rate^d^	76.25	66.80	67.47	76.24	5.636	0.952	0.954	0.112
Post-insemination								
Rectal temperature, °C	38.51	38.44	38.48	38.66	0.134	0.676	0.481	0.325
Respiration rate^d^	20.13	23.09	20.88	18.25	1.882	0.013	< 0.001	0.247

^a^For the lactation period, measurements were taken on day 18 of lactation between 16:00 and 18:00 h. For the post-insemination period, measurements were taken on day 12 post-insemination between 16:00 and 18:00 h

^b^The statistical analysis tested for main effects of dietary betaine, parity group, and their interaction. Sow was used as random effect

^c^Each value represents the least squares mean of 11 sows (44 sows total). For the lactation period, the average temperature, humidity, and heat index of the farrowing room were 28.1 °C (range of 27.3 to 28.7 °C), 72.9% (range of 68.3% to 77.4%) and 31.2 °C (range of 30.0 to 32.5 °C), respectively. Sows were above an environmental temperature and heat index of 26 °C for 100% of the time when measurements were taken. During the post-weaning period, environmental temperature, humidity, and heat index were 27.2 °C (range of 24.2 to 29.2 °C), 63.4% (range of 53.7% to 75%), and 28.7 °C (range of 25.1 to 31.2 °C), respectively. Sows experienced an environmental temperature and heat index over 26 °C for 91.3% and 94.5% of the time

^d^Respiration rate was measured as the number of flank movements per min

^e^Each value represents the least squares mean of 16 sows (64 sows total). For the lactation period, average temperature, humidity, and heat index of the farrowing room were 26.8 °C (range of 22.8 to 29.7 °C), 61.8% (range of 47.2% to 78.4%), and 28.2 °C (range of 24.4 to 32.7 °C), respectively. Sows were above an environmental temperature and heat index of 26 °C for 72.0% and 83.7% of the time. During the post-weaning period, the average temperature, humidity, and heat index of the breeding barn were 24.6 °C (range of 22.9 to 27.2 °C), 67.7% (range of 49.7% to 79.8%), and 26.0 °C (range of 23.8 to 29.4 °C). Sows experience environmental temperatures and a heat index of over 26 °C for 22.2% and 38.9% of the time, respectively

Following the lactation period, young sows (P1 and P2) tended to have greater weaning to estrus interval (*P* = 0.068) than mature sows (P3+) (Table 5). Supplementation of betaine post-weaning tended to reduce (*P* = 0.054) the weaning to estrus interval. A greater number of P3+ sows were bred within 7 and 14 days after weaning (bred within 7 and 14 days:weaned) and overall (bred:weaned) (*P* ≤ 0.037). Betaine supplementation post-weaning increased (*P* = 0.055) the number of sows bred within 7 days (bred within 7 days:weaned). Supplementation of betaine during lactation to P1 and P2 sows reduced the number of sows bred (bred:weaned), but did not affect P3+ sows (interaction, *P* = 0.004). Supplementation of betaine during lactation to P1 and P2 sows reduced the number of sows that returned to estrus after being bred (returns:bred within 14 days), but betaine during lactation to P3+ sows increased the number of sow that returned to estrus after being bred (interaction, *P* = 0.058). Supplementation of betaine during the post-weaning period tended to reduce the number of sows that farrowed (*P* = 0.060) regardless of the parity group. More P1 and P2 sows tended (*P* = 0.098) to be sold as cull sows.

**Table 5:** Effect of betaine supplementation on subsequent reproductive performance of sows during the summer months (Exp. 1)^d^

	Parity 1 and 2				Parity 3+				SEM	*P*-values^e^		
	Betaine in lactation diets, %											
	0	0	0.2	0.2	0	0	0.2	0.2		Parity group	Betaine lactation	Betaine post- weaning
	Betaine in post-weaning diets, %											
Item	0	0.2	0	0.2	0	0.2	0	0.2				
Sows, *n*	76	83	83	80	85	80	80	82				
Days to estrus	6.73	6.17	7.94	5.79	5.82	5.64	6.24	5.43	0.674	0.068	0.587	0.054
Bred within 7 days:weaned	0.842	0.916	0.783	0.850	0.894	0.913	0.888	0.927	0.036	0.026	0.256	0.055
Bred within 14 days:weaned	0.868	0.928	0.807	0.863	0.918	0.913	0.900	0.939	0.034	0.037	0.227	0.127
Overall bred:weaned^f^	0.974	1.000	0.940	0.913	0.988	0.975	1.000	0.988	0.018	0.015	0.059	0.606
Returns:bred within 14 days^g^	0.136	0.169	0.119	0.087	0.038	0.123	0.097	0.169	0.038	0.434	0.959	0.145
Farrowed:weaned^h^	0.921	0.880	0.855	0.825	0.953	0.875	0.925	0.890	0.035	0.098	0.175	0.060
Cull:weaned	0.053	0.108	0.072	0.138	0.047	0.075	0.063	0.049	0.029	0.098	0.648	0.104
Total pigs born^i^	12.55^b^	13.33^a,b^	13.39^a,b^	12.35^b^	14.17^a^	13.30^a,b^	14.46^a^	14.00^a^	0.462	0.001	0.511	0.224
Pigs born alive^j^	11.94^b,c^	12.75^a,b,c^	12.84^ab^	11.56^c^	13.05^a,b^	12.59^a,b,c^	13.20^a^	13.00^a,b^	0.439	0.026	0.827	0.367
Stillborn pigs^k^	0.62	0.58	0.55	0.79	1.11	0.71	1.27	1.00	0.160	0.001	0.196	0.286
Mummies	0.129	0.172	0.159	0.180	0.117	0.129	0.214	0.164	0.061	0.927	0.327	0.880

^a,b,c^Means with different superscripts differ (*P* ≤ 0.05)

^d^Values represent least squares means of n sows

^e^The statistical analysis tested for main effects of betaine supplementation during lactation, post-weaning, or both, parity group and their interactions. The group of placement (1 to 30) was used as the random effect in the analysis of days to estrus, total pigs born, pigs born alive, stillborn pigs, and mummies

^f^Lactation and parity group interaction (*P* = 0.004)

^g^Lactation and parity group interaction (*P* = 0.058)

^h^Sows that were weaned and farrowed regardless of the day that were bred

^i^Three factor interaction (*P* = 0.087)

^j^Three factor interaction (*P* = 0.057)

^k^Post-weaning and parity group interaction (*P* = 0.051)

Mature sows (P3+) had a greater number of total pigs born, pigs born alive and stillborn pigs than P1 and P2 sows (*P* ≤ 0.026). A tendency for a three-factor interaction was observed for the total number of pigs born (*P* = 0.087) and pigs born alive (*P* = 0.057). For P1 and P2 sows, despite the fact that total pigs born and pigs born alive were not different among treatments, the greatest number of pigs were observed when sows received betaine during lactation and the lowest number of pigs when sows received betaine in both periods. For P3+ sows, despite the fact that total number of pigs born and pigs born alive were not different among treatments, the greatest number of pigs were observed when sows received betaine during lactation and the lowest number of pigs when sows received betaine during the post-weaning period.

An exploratory analysis was conducted to further separate the parity groups. Sows were clustered into three parity groups: parity 1 (P1), parity 2 and 3 (P2 and P3), and parity 4 or greater (P4+) to determine effects of supplemental betaine (Table 6). For P1 sows, an interaction of betaine supplementation during lactation and betaine supplementation during the post-weaning period was observed for the number of total pigs born (*P* = 0.025) and pigs born alive (*P* = 0.007). Supplementation of betaine to P1 sows during the post-weaning period increased total number of pigs born and pigs born alive. However, supplementation of betaine during lactation and subsequently in the post-weaning period reduced total number of pigs born and pigs born alive. For P2 and P3 sows, betaine supplementation during lactation or the post-weaning period did not affect days to estrus, farrowing rate, total pigs born or pigs born alive. For P4+, supplementation of betaine during lactation increased the total number of pigs born (*P* = 0.026) and supplementation of betaine during the post-weaning period reduced the total number of pigs born (*P* = 0.014). Supplementation of betaine during the post-weaning period tended to reduce pigs born alive to P4+ sows (*P* = 0.084).

**Table 6:** Exploratory analysis of dietary betaine on subsequent reproductive performance during summer months (Exp. 1)^c^

Item	Betaine in lactation diets, %				SEM	*P*-values^d^		
	0	0	0.2	0.2		Betaine lactation	Betaine post-weaning	Interaction
	Betaine in post-weaning diets, %							
	0	0.2	0	0.2				
Parity 1, *n*	39	44	44	42				
Days to estrus	5.78	6.82	7.80	5.19	0.960	0.843	0.415	0.061
Farrowed:weaned^e^	0.923	0.841	0.818	0.833	0.054	0.327	0.555	0.328
Total pigs born	12.32^a,b^	13.82^a^	13.40^a,b^	12.18^b^	0.598	0.638	0.821	0.025
Pigs born alive	11.61^b^	13.33^a^	12.93^a,b^	11.38^b^	0.598	0.599	0.888	0.007
Parity 2 and 3, *n*	71	71	71	72				
Days to estrus	7.21	5.69	7.16	6.66	0.815	0.569	0.216	0.526
Farrowed:weaned^e^	0.929	0.915	0.915	0.847	0.035	0.245	0.245	0.444
Total pigs born	13.42	13.20	13.41	13.01	0.541	0.848	0.550	0.864
Pigs born alive	12.58	12.56	12.85	12.25	0.515	0.969	0.521	0.540
Parity 4, 5 and 6, *n*	51	48	48	48				
Days to estrus	5.28	5.56	6.25	4.51	0.685	0.957	0.242	0.107
Farrowed:weaned^e^	0.961	0.854	0.916	0.896	0.041	0.977	0.127	0.303
Total pigs born	14.15	12.90	15.37	14.02	0.585	0.026	0.014	0.927
Pigs born alive	13.07	12.06	13.59	12.88	0.552	0.178	0.084	0.768

^a,b^Means with different superscripts differ (*P* ≤ 0.05)

^c^Values represent least squares means of *n* sows

^d^The statistical analysis tested for main effects of betaine supplementation during lactation and/or post-weaning, and their interaction. The group of placement (1 to 30) was used as the random effect in the analysis of total pigs born and pigs born alive

^e^Sows that were weaned and farrowed regardless of the day that were bred

### Exp. 2

The average temperature of the farrowing room during the lactation period was 23.1 ± 2.7 °C (minimum of 15.1 and maximum of 33.7 °C) and relative humidity was 59.3% ± 13.5% (minimum of 2.7% and maximum of 95.2%), resulting in a heat index of 25.1 °C (minimum of 16.2 and maximum of 37.2 °C). Sows experienced an environmental temperature over 26 °C for 16.3% of the time and a heat index of over 26 °C for 20.6% of the time. The average temperature of the breeding barn (during the post-weaning period until 35 days post-insemination) was 23.9 ± 2.0 °C (minimum of 17.4 and maximum of 33.5 °C) and humidity was 69.9% ± 12.3% (minimum was 8.4% and maximum was 93.5%). The heat index averaged 25.1 °C (minimum of 18.3 and maximum of 41.7 °C). Sows were above an environmental temperature of 26 °C for 15.2% of the time and above a heat index of 26 °C for 21.1% of the time.

Mature sows (P3+) had greater (*P* < 0.001) BW at placement, after farrowing, and at day 21 of lactation (Table 7). During the lactation period, P3+ sows gained BW, while P1 and P2 sows had BW losses (*P* < 0.001). Average daily feed intake and sow gain:feed were greater for P3+ sows (*P* < 0.001). Litter gain was greater for P3+ sows (*P* < 0.001), but there were no differences in the number of pigs weaned or piglet mortality due to parity group (*P* ≥ 0.816). Supplementation of betaine during the lactation period did not affect sow or litter performance (*P* ≥ 0.155).

**Table 7:** Effect of betaine fed during lactation on sow and litter performance during non-summer months (Exp. 2)^a^

Item	Parity 1 and 2		Parity 3+		SEM	*P*-values^b^	
	Betaine, %						
	0	0.2	0	0.2		Betaine	Parity group
Sows, *n*	150	150	163	164			
BW at placement, kg^c^	225.8	223.6	276.7	275.7	2.337	0.469	< 0.001
BW at farrowing, kg^d^	206.4	204.5	252.3	252.4	2.289	0.666	< 0.001
BW at day 21, kg	199.5	196.5	255.0	254.2	2.245	0.348	< 0.001
BW change, kg	−7.1	−7.9	2.0	1.8	1.428	0.698	< 0.001
Sow BW gain, kg/d	−0.340	−0.376	0.096	0.087	0.068	0.698	< 0.001
Lactation length, d	22.96	23.18	23.16	23.27	0.113	0.075	0.110
Feed intake, kg/d	4.31	4.24	5.61	5.47	0.101	0.155	< 0.001
Sow gain:feed^e^	0.412	0.401	0.444	0.486	0.015	0.203	< 0.001
Litters, *n*^f^	81	84	93	94			
Litter weight after cross-fostering, kg^g^	17.21	17.10	19.40	19.29	0.287	0.687	< 0.001
Litter weight on day 21, kg	62.0	60.7	69.6	69.9	1.304	0.699	< 0.001
Litter gain, kg	45.3	43.6	50.2	50.7	1.253	0.622	< 0.001
Pigs weaned per litter	10.79	10.76	10.78	10.72	0.100	0.687	0.816
Piglet mortality, %	10.10	10.27	10.14	10.62	0.837	0.704	0.816
No-value pigs, %^h^	6.12	5.17	3.85	4.40	0.997	0.829	0.104

^a^Values represent least squares means of *n* sows. This Experiment was conducted from February to June

^b^The statistical analysis tested for main effects of betaine supplementation, parity group and their interactions. Group of placement (1 to 28) was used as the random effect. No significant interactions were detected (*P* ≥ 0.21)

^c^Sow body weight (BW) was measured prior to entering the farrowing room (112 ± 2 day of gestation)

^d^Sow BW after farrowing was calculated using equations of Walker and Young [20]

^e^Sow gain:feed was calculated as sow BW gain or loss plus total litter gain divided by total feed intake during lactation

^f^A subset of 352 sows was used to measure litter performance (group 1 to 16). Data were collected from February to April

^g^Litters were standardized to 12 pigs per litter

^h^No value pigs consisted of pigs weighing less than 3.62 kg at day 21 of lactation

The average daily temperature of the farrowing room was and 26.8 ± 1.5 °C (range of 22.8 to 29.7 °C), humidity was 61.8% (range of 47.2% to 78.4%, and heat index of 28.2 °C (range of 24.4 to 32.7 °C) when rectal temperature and respiration rate were measured in the subset of sows. Sows were above an environmental temperature and heat index of 26 °C for 72.0 and 83.7% of the time. During the post-weaning period, the average temperature of the breeding barn was 24.6 °C (range of 22.9 to 27.2 °C), humidity was 67.7% (range of 49.7% to 79.8%), and heat index was 26.0 °C (range of 23.8 to 29.4 °C). Sows experience environmental temperatures and a heat index of over 26 °C for 22.2% and 38.9% of the time, respectively. Betaine supplementation in lactation did not affect (*P* ≥ 0.772) rectal temperature or respiration rate, but respiration rate was decreased (*P* = 0.013) when betaine was supplemented post-insemination (Table 4).

A tendency for a three-factor interaction was observed for days to estrus (*P* = 0.09) (Table 8). Supplementation of betaine only during the post-weaning period increased the numbers of days to estrus in P1 and P2 sows compared to P1 and P2 sows fed betaine in both periods (lactation and post-weaning) without impacting P3+ sows. As an overall effect, betaine supplementation during lactation tended to reduce days to estrus (*P* = 0.077). Supplementation of betaine during the post-weaning period to P1 and P2 sows reduced the number of sows bred (bred:weaned) (interaction, *P* = 0.079), but did not affect P3+ sows.

**Table 8:** Effect of betaine supplementation on subsequent reproductive performance of sows during non-summer months (Exp. 2)^c^

Item	Parity 1 and 2				Parity 3+				SEM	*P*-values^d^		
	Betaine in lactation diets, %											
	0	0	0.2	0.2	0	0	0.2	0.2		Parity group	Betaine lactation	Betaine post- weaning
	Betaine in post-weaning diets, %											
	0	0.2	0	0.2	0	0.2	0	0.2				
Sows, *n*	77	73	74	76	81	82	83	81				
Days to estrus^e^	6.67^a,b^	8.19^a^	6.83^a,b^	5.76^b^	8.21^a^	6.93^a,b^	7.26^a,b^	6.70^a,b^	0.752	0.401	0.077	0.473
Bred within 7 days:weaned	0.792	0.740	0.811	0.803	0.679	0.780	0.735	0.765	0.048	0.172	0.370	0.601
Bred within 14 days:weaned	0.831	0.753	0.838	0.829	0.778	0.817	0.855	0.790	0.044	0.930	0.290	0.369
Overall bred:weaned^f^	0.987	0.918	0.973	0.921	1.000	1.000	0.988	0.963	0.019	0.005	0.268	0.008
Returns:bred within 14 days^g^	0.109	0.109	0.081	0.095	0.143	0.119	0.239	0.219	0.044	0.008	0.214	0.809
Farrowed:weaned^g,h^	0.870	0.822	0.865	0.803	0.963	0.915	0.831	0.802	0.039	0.169	0.015	0.089
Cull:weaned^i^	0.104	0.153	0.108	0.171	0.025	0.061	0.120	0.148	0.035	0.069	0.040	0.079
Total born	12.54	12.37	13.53	12.19	14.49	13.77	14.00	13.66	0.441	< 0.001	0.868	0.040
Born alive	12.13	11.85	12.56	11.72	13.54	12.76	13.05	12.88	0.412	0.001	0.954	0.075
Still born	0.41	0.53	0.84	0.47	0.88	0.91	0.83	0.78	0.126	0.001	0.620	0.448
Mummies	0.222	0.132	0.143	0.158	0.305	0.214	0.322	0.138	0.064	0.073	0.536	0.054

^a,b^Means with different superscripts differ (*P* ≤ 0.05)

^c^Values represent least squares means of *n* sows

^d^The statistical analysis tested for main effects of betaine supplementation during lactation and/or post-weaning, parity group and their interactions. The group of placement (1 to 28) was used as the random effect in the analysis of days to estrus, total born, born alive, still born, and mummies

^e^Three way interaction (*P* = 0.09)

^f^Post-weaning and parity group interaction (*P* = 0.079)

^g^Lactation and parity group interaction (*P* ≤ 0.05)

^h^Sows that were weaned and farrowed regardless of the day that were bred

^i^Lactation and parity group interaction (*P* = 0.108)

A greater number of P3+ sows returned to estrus (*P* = 0.008) after being bred (returns:bred with 14 days) compared to P1 and P2 sows. In addition, supplementation of betaine during lactation to P3+ sows increased the number of sows that returned to estrus after first insemination (returns:bred within 14 days; interaction, *P* = 0.040), but did not affect P1 and P2 sows. Supplementation of betaine during lactation to P3+ sows reduced the number of sows that farrowed (interaction, *P* = 0.047), but did not affect P1 and P2 sows. The cull rate tended to be higher for P1 and P2 sows (*P* = 0.069) compared to P3+ sows. However, among P3+ sows, the group that received betaine during lactation had a higher cull rate (interaction, *P* = 0.108).

Mature sows (P3+) had a greater number of total pigs born, pigs born alive, stillborn pigs (*P* ≤ 0.001), and tended to have more mummies (*P* = 0.073) compared to P1 and P2 sows. Supplementation of betaine in the post-weaning period reduced total number of pigs born (*P* = 0.040) and tended to reduce pigs born alive (*P* = 0.075) and mummies (*P* = 0.054), regardless of parity group.

## Discussion

Dietary supplementation of betaine has been shown to improve the subsequent reproductive performance of sows when fed during lactation [9, 10] and gestation [13] in the summer months. In the present studies, we evaluated betaine supplementation during lactation and the post-weaning until day 35 post-insemination period to determine the optimum time frame for betaine supplementation. In addition, we evaluated which parity of sows were more responsive to betaine supplementation, based on previous data that suggested that mature sows had a greater response than young sows [12]. Two studies were conducted, one during the summer months and one during non-summer months to determine if the effect of betaine was more pronounced during the summer season or could also be detected during the non-summer season.

The diets in the present studies were formulated to provide enough choline, methionine, and folic acid to supply sufficient quantities of methyl donors in order to specifically determine the impact of the osmolyte properties of betaine. The choline requirement for lactating sows is 5.25 g/d and for gestating sows is 2.3 g/d [18]. During the lactation period, sows were provided with 8.74 g/d of choline and during the post-weaning period with 2.5 g/d of choline. Methionine requirements for lactating and gestating sows are 0.22% and 0.12% of standardized ileal digestible (SID) methionine, respectively [18]. Diets in the present studies were formulated to provide 0.28% and 0.18% of SID methionine, respectively. The concentration of betaine evaluated in the present experiments were based on Campbell and Virtanen [12], who reported that betaine supplementation at 0.2% to diets meeting all nutrient requirements increased the number of pigs in the subsequent cycle by 1 pig, whereas betaine supplemented at 0.4% reduced sow feed intake.

The analyzed concentrations of betaine in the experimental diets showed an average of 0.216% and 0.222% additional betaine in supplemented lactation and gestation diets, respectively, which is consistent with the target of 0.20% supplemental betaine. Betaine concentrations were higher in the gestation diets compared to the lactation diets because of higher endogenous concentrations of betaine in the control gestation diet, which is likely related to the relatively high concentration of betaine in wheat middlings compared to very low levels in corn [21].

Exp. 1 was conducted during the summer months. The average temperature in the farrowing room was 2.3 °C greater and humidity was 13.2% greater than the respective averages recorded in the non-summer experiment. Moreover, sows experienced a temperature of 26 °C or greater 48.9% (62.3% for heat index) of the time during the lactation period in the summer compared to 16.3% (20.6% for heat index) in the non-summer experiment, indicating significant exposure to heat stress. During the breeding period, environmental temperatures and humidity were relatively similar when evaluating averages and sows experienced slightly more time exposed to temperatures over 26 °C during the summer (19.1% and 28.0% for temperature and heat index, respectively) compared to the non-summer months (16.3% and 21.1% for temperature and heat index, respectively). The temperature exposure differences in the summer compared to non-summer, especially in lactation, are practically relevant and consistent with other studies evaluating the impact of betaine in sows during heat stress. In spite of these differences in heat exposure, respiration rate and rectal temperatures were not markedly different between the summer and non-summer months.

In the summer experiment, sows were 13 kg heavier when entering the farrowing rooms than the sows in the non-summer experiment. Nonetheless, BW on day 21 was similar in both experiments. Therefore, BW losses were greater in the summer experiment, which was associated with a reduction in feed intake of 14%. Surprisingly, in the summer experiment, litter gain was 13% greater and sows weaned 0.2 more pigs compared to the non-summer experiment.

Supplementation of betaine during lactation in the summer experiment reduced feed intake, but the reduction in feed intake was not observed in the non-summer study. It should be noted that feed intake of sows during the non-summer experiment was greater than that of sows during the summer experiment, thus betaine intake was actually greater for sows in the non-summer experiment and may have contributed to differences between experiments. Reduction in feed intake in lactating sows has been observed when natural betaine was added at 0.2% [9] and at 0.4% [12]. However, Cabezón et al. [11] reported an increase in feed intake when 0.3% of betaine hydrochloride (70.7% betaine) was added to diets of lactating sows, specifically in parity 2 sows. In another study, Cabezón et al. [22] reported increased feed intake in parity 1 and 2 sows when betaine was supplemented at 0.3% as betaine hydrochloride, but feed intake was not affected by betaine in parity 3 to 5 or parity 6+ sows. In a meta-analysis, Sales [23] reported a mean effect side (Hedges’s g) of − 0.155 (95% confidence interval was − 0.352 to 0.043) indicating a negative association of feed intake and betaine supplementation in finishing pigs; however, data showed a substantial amount of heterogeneity, indicating that responses between studies were highly variable. In the present studies, betaine supplementation during lactation did not affect litter performance, regardless of the season. These results are in agreement with Campbell and Virtanen [12] who did not observe an improvement in litter gain due to dietary betaine. Nonetheless, Ramis et al. [9] and Greiner et al. [10] reported an improvement in litter gain and no differences in the number of pigs weaned when betaine was supplemented.

Interestingly, sows weaned in the non-summer presented signs of estrus 0.8 days later than sows weaned in the summer months. Supplementation of betaine during lactation consistently reduced weaning to estrus interval [9–11]. In the summer experiment, betaine supplementation during lactation did not impact the number of days to estrus, however, betaine supplementation post-weaning reduced days to estrus. In the non-summer experiment, betaine supplementation during lactation reduced the number of days to estrus; however, betaine supplementation post-weaning did not affect days to estrus.

In both experiments, supplementation of betaine during the post-weaning period reduced farrowing rate. There is no prior research available on the impacts of feeding betaine specifically during the post-weaning period until 35 days post-insemination to contrast the findings in the present study. However, a potential cause could be related to the total concentration of betaine present in the diets fed in the post-weaning period. The diets contained 0.489% and 0.508% of total betaine for Exp. 1 and 2, respectively. These high levels could have negatively affected sow farrowing rate, although the specific mechanisms underlying this effect are not understood. Campbell and Virtanen [12] reported a reduction in farrowing rate when lactating P1 sows were fed betaine at 0.4% (83% farrowing rate), but not when fed 0, 0.1% or 0.2% (89%, 94% and 91% farrowing rate, respectively). In contrast, other studies have not reported a decrease in farrowing rate due to betaine supplementation during lactation [9–11].

The greatest benefit of using betaine in diets of sows has been reported for subsequent litter size. Campbell and Virtanen [12] reported that P2+ sows had an increased total number of pigs born by 2.3 piglets when betaine was fed, while parity 1 sows did not show an increase. Van Wettere et al. [13] reported an increase in subsequent litter size when betaine (7.6 to 9 g/d per day) was fed to mature sows during gestation by 1.5 additional piglets. In the current studies, we did not observe an increase in litter size when betaine was supplemented in the non-summer experiment. Nonetheless, in the summer experiment betaine supplementation during lactation to P4+ sows increased total number of pigs born by 1.2 piglets, but supplementation of betaine in the post-weaning period decreased total number of pigs born by 1.3 piglets.

The mechanism by which betaine may reduce the weaning to estrus interval and increase litter size is not yet fully understood. Supplemental betaine may improve follicular growth in sows as indicated by increased follicular size measured by transrectal ultrasonography [24]. Studies in mice have shown a possible mechanism of betaine increasing embryo development and survival. Transporters of betaine are active during the 2- and 4-cell stage, approximately up to 30 h after fertilization [25]. During these stages, betaine accumulated in the rodent embryo and potentially served as an osmoprotectant prior to implantation in the initial cellular division and later as a methyl donor in the blastocyst [14]. In addition, betaine serves as a methyl donor, regenerating methionine from homocysteine. High levels of homocysteine have been shown to be associated with pregnancy complications and early pregnancy loss in women [26]. The enzyme betaine-homocysteine methyltransferase was thought to be unique to the liver [27]. Recent studies have shown that this pathway also exists in the blastocyst [15, 28]. Lee et al. [15] found that impaired activity of betaine-homocysteine methyltransferase in the embryo causes embryo reabsorption.

## Conclusions

No beneficial effects were observed when betaine was supplemented to sows during the non-summer months. Supplementation of betaine during the summer in the lactation period to parity 4+ sows increased the subsequent litter size. Supplementation of betaine during the summer in the post-weaning period to 35 days post insemination reduced wean-to-estrus interval and increased the total number of pigs born for parity 1 sows. However, feeding betaine in the post-weaning period reduced farrowing rate and reduced the total number of pigs born in parity 4+ sows. Further research is needed to evaluate supplementation of betaine only during the weaning until breeding period to capture the benefits of reducing wean-to-estrus interval without affecting farrowing rate. In addition, the detrimental effects in feed intake and farrowing rate may be correlated and related to the level of betaine in the diet. Evaluating the optimal level of endogenous and supplemental betaine in the diet of sows will be essential to produce more consistent results.

## Data Availability

The dataset analyzed in the present study can be made available from the corresponding author upon reasonable request.
